# Parkinson’s Disease and Its Dermatological Associations: Is Your Skin Whispering You a Diagnosis?

**DOI:** 10.7759/cureus.9933

**Published:** 2020-08-22

**Authors:** Puja Shah, Prem Raj Sagar, Norah Alhumaidi, Vijaya Chaitanya Bollampally, Bilal Haider Malik

**Affiliations:** 1 Internal Medicine, California Institute of Behavioral Neurosciences & Psychology, Fairfield, USA; 2 Neurological Surgery, California Institute of Behavioral Neurosciences & Psychology, Fairfield, USA

**Keywords:** skin, parkinson's disease, melanoma, seborrheic dermatitis, bullous pemphigoid, rosacea

## Abstract

Parkinson's disease (PD) is a neurodegenerative disease characterized clinically by the triad of resting tremor, rigidity, and bradykinesia. Although PD is primarily known for motor disturbance, 98.6% of patients experience one or more non-motor symptoms at all stages of the disease. Dermatological disorders are discussed as common non-motor associations of PD since the 20th century. Many studies have shown that patients of PD are predisposed to skin disorders. This article is a traditional review done to analyze the association between PD and its dermatological manifestations. We did a literature search using six keywords in the PubMed database and took the relevant articles published in the last 10 years. We reviewed more than 100 articles, which also included animal studies. On meticulous review, we observed an increased incidence of certain skin disorders like seborrheic dermatitis, bullous pemphigoid, rosacea, and melanoma in patients of PD. These disorders share either common risk factors or underlying mechanisms revolving around genetics, immunology, inflammation, and pathophysiology of PD, but the exact causation yet seems obscured. We believe that this opens a horizon for more research in the link between the skin and nervous system. We also emphasize that the dermatologists, neurologists and general physicians should address the cutaneous disorders in PD timely, educate their patients, help them lessen the psychosocial distress, and improve their quality of life.

## Introduction and background

The second most common neurodegenerative disease is Parkinson's disease (PD) [1]. It had a worldwide burden of 2.5 million in 1990, which increased to 6.1 million in 2016. The incidence is 1.4 times more in males as compared to females after the fifth decade of life. There is an increase in the burden of PD with increasing age, decreasing smoking and improved socio-demographic index [2]. 

PD is primarily a motor disorder, but it also manifests with one or more non-motor symptoms in 98.6% of patients at all stages of the disease [3]. It is defined as a clinical triad of resting tremor, rigidity, and bradykinesia [4]. However, non-motor symptoms may also precede or appear during the disease course, along with neurological symptoms. This observation is leading to a change in the definition of PD, which no longer remains limited to the above triad [4]. The non-motor symptoms of PD include cognitive dysfunction, mood disorders, sleep disorders, gastrointestinal disorders, urinary disorders, autonomic dysfunction, and dermatological disorders [3].

There is a loss of dopaminergic neurons in the substantia nigra in PD. It explains the basis of using levodopa (L-dopa) in the treatment. Nevertheless, the lack of disease-modifying therapy shows that the understanding of PD pathogenesis is still an under-explored arena. There are several proposed theories like misfolding of alpha-synuclein, neuroinflammation, mitochondrial dysfunction, neurotransmitter driven alteration of brain neuronal networks [5]. Several genetic mutations, like Parkin (PARK2) and PTEN-induced kinase 1 (PINK1) (PARK6) genes, are also implicated in many cases [5]. There is widespread extranigral involvement all across the human body from the skin, gastrointestinal tract to the peripheral nerves in PD [3, 4].

Dermatological disorders are one of the common non-motor symptoms of PD discussed since the 20th century [5]. The associated skin problems are not recognized and are often underestimated while planning the strategy for diagnosis and treatment. This article will focus on the dermatological associations that can precede before or present during the motor symptoms of PD, which must be addressed by the treating physician, potentially improving the patient's quality of life. Exploring the increased incidence of certain skin disorders in PD may fill the missing links in the pathogenesis of PD. This article intends to review most of the recent articles published on PubMed, which shows the relation between PD and skin disorders, and also, the references of those articles are accessed to reveal additional relevant studies.

## Review

Method

Literature was searched in PubMed using regular keywords and Medical Subject Headings (MeSH) keywords and data collection was done. Table 1 shows regular keywords and MeSH keywords for literature search.

**Table 1:** Regular keywords and MeSH keywords for literature search MeSH: Medical Subject Headings

Regular Keyword	Number of Results	MeSH Keyword	Number of Results
Skin	792,954	Skin	220,697
Melanoma	132,696	Melanoma	93,300
Parkinson’s disease	110,684	Parkinson disease	64,695
Bullous Pemphigoid	5,450	Parkinson disease and Skin	164
Rosacea	4,005	Parkinson disease and Melanoma	103
Seborrheic dermatitis	3,394		
Parkinson’s disease, Skin	934		
Parkinson’s disease, Melanoma	238		
Parkinson’s disease, Neurodermatology	62		
Parkinson’s disease, Bullous pemphigoid	43		
Parkinson’s disease, Seborrheic dermatitis	42		
Neurodermatology	25		
Parkinson’s disease, Rosacea	21		

Studies were selected after applying the following inclusion criterion: paper published within the past 10 years. No exclusion criteria were used.

Results

Table 2 shows the total number of articles after applying inclusion criteria.

**Table 2:** Total number of articles after applying inclusion criterion MeSH: Medical Subject Headings

Regular Keyword	Number of results after inclusion criterion	MeSH Keyword	Number of results after inclusion criterion
Skin	269,286	Skin	57,449
Melanoma	56,137	Melanoma	31,797
Parkinson’s disease	56,987	Parkinson disease	27,846
Bullous Pemphigoid	1,943	Parkinson disease and Skin	74
Rosacea	1,763	Parkinson disease and Melanoma	45
Seborrheic dermatitis	747		
Neurodermatology	11		
Parkinson’s disease, Skin	539		
Parkinson’s disease, Melanoma	132		
Parkinson’s disease, Neurodermatology	61		
Parkinson’s disease, Bullous Pemphigoid	35		
Parkinson’s disease, Seborrheic dermatitis	11		
Parkinson’s disease, Rosacea	17		

A cumulative of more than 100 articles were selected in writing this literature review. Additional studies were identified from manual searches of references in the retrieved articles.

Discussion

Seborrheic dermatitis

Seborrheic dermatitis (SD) is a chronic inflammatory skin disorder affecting sebum rich areas of the face, scalp, upper back, and chest, presenting predominantly as red scaly rashes. About 52-59% of PD patients, as opposed to 3% in the general population, suffer from SD leading to significant psychosocial distress [6].

*Malassezia *species (*M. restrica *and *M. globosa*) are the fungi present on the skin of patients of SD. The fact that antifungal works well against SD suggest that this commensal yeast has a role in the disease process. Malassezia usually proliferates in sebum rich areas. According to a study by Arsenijevic et al., PD also promotes the production of sebum on the skin [6]. The dopamine deficiency causes an inadequacy of melanocyte-stimulating hormone (MSH) inhibiting factor. This inadequacy increases the level of α-MSH, which results in a surge of sebum production. Similarly, reduced facial mobility in PD results in poor hygiene, contributing to more sebum accumulation [6-8]. This correlation gives a clue to any relationship, if present, between Malassezia and PD. A study by Kohn SR et al. shows an improvement of seborrheic dermatitis after starting L-dopa therapy in PD [9]. However, some other studies depict no such improvement [10]. A retrospective study by Taner et al. endorses SD as a premotor feature of PD. According to this study, SD increases the risk of being diagnosed with PD many years later [11]. These caveats in studies contradicting each other cannot establish Malassezia entirely as an association between SD and PD.

Many studies revolve around the role of CD4 T cells in SD, PD, and Malassezia growth. The T-helper cell (TH1) is important in the control of Malassezia and this is explained by the use of immune modulators in the treatment of SD [12]. A study by Lally A et al. shows that anti-T cell drugs (azathioprine and cyclosporine) are a major risk factor for SD [13]. Similarly, there is an association of low CD4 T cell count in PD and a weak TH1 response against Malassezia in patients of PD having concomitant SD [14,15]. Another study suggests that reduced facial mobility in PD causes a decrease in lymph flow, restricting the access of CD4 T cells to reach skin [7]. These studies can be linked by the fact that acquired immune deficiency syndrome (AIDS), which have weak CD4 T cell response, shows an increased risk of SD as well as increased incidence of parkinsonism in a young individual [7]. However, the response of SD to anti-retroviral therapy is variable in patients of AIDS. So we can only assume that SD and PD can be linked with weak TH1 response. More studies are required to fully support that.

PD and SD share common risk factors or underlying mechanisms but the exact pathophysiology showing one causes the other is yet to be determined. As this is a chronic inflammatory skin condition patients should be educated to focus on limiting the symptoms, rather than cure, and enhance the quality of life. Diagnosis of SD is mainly clinical and various antifungal agents, keratolytic, corticosteroids and immunomodulators can be used [12].

Rosacea

Rosacea is a benign chronic skin disorder with a multifactorial cause. There are four major clinical subtypes: erythematotelangiectatic (ETR), papulopustular (PPR), phymatous (PhR), and ocular. This inflammatory condition presents with a constellation of symptoms like flushing, persistent redness, inflammatory papules, pustules, telangiectasia, and hypertrophy of sebaceous glands of the nose. It has a prevalence of approximately 2% to 22% in the white population. As it predominantly affects the face, about 75% of rosacea patients have low self-esteem stressing the psychosocial impact of this condition [16].

In 2001 an observational study consisting of 70 PD patients showed surprising data. About 18.8% of them had rosacea, and 31.9% of them had flushing symptoms [17]. Later a large epidemiological study to examine the association between rosacea and PD was done by Egeberg alexander et al. It was a Danish cohort study done on 5.4 million adults observed for 15 years (1997-2011). There was a two-times increase in the risk of PD among patients diagnosed with rosacea than those who were not, and the incidence of PD was 2.4 years earlier than the reference population. The increased risk of PD was seen mostly in the ocular subtype of rosacea. There was a 2% decrease in the risk of PD in those taking the tetracyclines prescription regardless of rosacea diagnosis [18]. Another large retrospective study in the US using the electronic medical record of 803,005 adult patients (17,682 with rosacea diagnosis) followed over more than five years showed an increased risk of PD in rosacea patients (OR=1.39;95% CI:1.04-1.85; p<0.02).The age at rosacea diagnosis was higher in patients who ultimately got PD than those rosacea patients who did not get PD. Out of rosacea patients, those exposed to tetracycline showed no relation between rosacea and PD, whereas those not exposed to tetracyclines showed the relation [19]. Many other studies hypothesize the possible mechanisms linking the PD and rosacea [20-22].

Rosacea has up-regulation of matrix metalloproteinase (MMP) enzyme, notably MMP-1, MMP-3, MMP-9, which has a role in tissue breakdown and repair [20]. Likewise, a mice model of PD also depicts the role of MMP-1, MMP-3 in cerebrospinal fluid (CSF) in dopaminergic inflammation, and neurodegeneration [21]. Another common link to both diseases is the association with Helicobacter pylori and small intestinal bacterial overgrowth (SIBO) [22]. PPR form of rosacea has shown an association with microorganisms [22]. Furthermore, there is also evidence of aggravation of neuronal deaths in patients with long term gastrointestinal infections by these organisms [22]. Treatment of these infections led to improved motor fluctuations in PD [23]. These studies show the microbes-gut-brain axis, which requires further exploration. Neurokinin B, which often accompanies the onset of rosacea, is also involved in the pathogenesis of PD [24]. For ages, doctors have been using tetracycline in the treatment of rosacea. These drugs offer neuroprotection delaying the onset of PD, as seen in the mouse PD model [25], and this finding is consistently seen in other studies [18,19].

Patients should avoid triggers of flushing, follow sun protection, and proper skincare. Therapeutic interventions for rosacea range from the use of topical metronidazole, topical azelaic acid, and topical doxycycline to laser therapies according to the severity of the disease [16].

Bullous pemphigoid

Bullous pemphigoid (BP) is an autoimmune bullous disease of the skin, mainly seen in the elderly. The median age of onset is 75 years, more commonly observed in women. Patients suffer from generalized pruritic bullous skin eruptions. This eruption is the result of the body’s humoral and cellular response against a component of hemidesmosomes, BP antigen 180 (BP180), and BP antigen 230 (BP230) [26].

The mortality rate of patients with BP seen in eastern and western cohorts is higher than that of the general population [27, 28]. Several co-morbidities accompany elderly BP patients, and neurological disorders are one of them [29]. The mean time interval by which neurological disorder precedes BP is 6.7 years, as observed in one of the systematic reviews [30]. Patients of BP are more likely to develop multiple sclerosis as neurological co-morbidity [29], while other commonly observed are dementia and Parkinson's disease [29]. Studies show that the presence of neurological co-morbidity gives a poor prognosis in patients of BP [27, 29]. Likewise, the presence of PD in patients of BP also increases their mortality [27, 28]. The neurological disorder thus appears as a significant prognostic factor in patients of BP. Many studies calculate PD to be three times more likely to be seen in patients suffering from BP than the general population [29, 30]. A meta-analysis by Lai YC et al. states that the patients of BP are more likely to have a neurological disorder, and among that for PD, Relative Risk (RR) = 3.42, 95% CI: 3.01-3.8 [29].

The revelation of auto-antigens BP180 and BP230 in central nervous system (CNS) apart from the skin discloses the epidemiological relation of BP with neurological diseases [31, 32]. We can postulate a cross-reactive immune response between neural and cutaneous antigens of neurodegeneration and neuroinflammation [33]. Circulating immunoglobulin G (IgG) antibodies were found in a subset of patients of PD directed against BP 180, but their role was doubtful as these anti neural antibodies exhibited no tropism to the skin, and neither did it cause BP like symptoms in them [34]. Psychiatric disorders like bipolar disorders, schizophrenia, personality disorders are also observed more frequently in BP patients, although their risk ratio is lower than the neurological disorders [35]. This association opens the horizon for further research on the role of autoimmunization against neural BP in the pathogenesis of BP.

Physicians should acknowledge the relation of neurological disorder in BP and the subsequent increase in mortality. This issue should be addressed in a therapeutic approach to decrease psychosocial stress.

Melanoma

Melanoma arises from the melanin-producing cells of the epidermis. The mortality in patients of Parkinson's disease from cancer is significantly lower than that of the general population except for melanoma [36]. The annual new cases of melanoma, in US whites, is predicted to rise from 70,000 in 2007-2011 to 116,000 in 2026-2031. A total of 21% of this data is attributed to population growth and aging [37].

A large epidemiological study conducted showed that (phase I) patients with PD were 3.8 times more likely to have preexisting melanoma as compared to controls. Whereas in phase II of study, melanoma patients had 4.2 times increased risk of developing PD. Surprisingly, patients who had melanoma without PD were 10.5 times more likely to die from metastatic melanoma compared to patients who had melanoma and PD [38]. Another study of the National Institutes of Health (NIH) Exploratory Trials in PD (NET-PD) Long-term Study 1 cohort also showed a similar risk of malignant melanoma, with a standardized event ratio of 3.6 (95% confidence interval, 2.2-5.6) [39]. Similarly, another large prospective study of 157,036 patients followed for 14-20 years concluded that the family history of melanoma in 1st-degree relative was associated with a higher risk of PD with a multi-variant relative risk of 1.85 [40].

There are many postulated theories for the association between PD and melanoma. One of them is levodopa therapy, which speculated that levodopa therapy increased the growth and metastatic spread of malignant melanoma in PD [41]. However many trials later, one of them, the deprenyl and tocopherol antioxidative therapy of parkinsonism (DATATOP) clinical trial, clarified the poor link between them, weakening this theory [42]. There is a defect in autophagy in PD, which results in the aggregation of proteins and damaged mitochondria [43]. This defective autophagy is also used by melanoma to escape the immune system [44]. Another theory is the role of α-synuclein in PD and melanoma. Under pathological stress, α-synuclein accumulates in the dopaminergic neurons. It forms intermediates with dopaquinone and other dopamine metabolites that cause the death of neurons [45]. Melanoma cells also express a high level of α-synuclein. These cells produce a low level of melanin due to inhibition of the activity of tyrosine hydroxylase enzyme [46]. Based on these clues, we can assume a possible explanation for an increase in the risk of melanoma in patients of PD. The patients of PD have increased α-synuclein in their melanocytes, which decreases melanin synthesis and thus increases the risk of melanoma in them [47]. There is also an increase in the risk of PD with decreasing darkness of hair colour. This observation highlights the role of pigmentation in PD and melanoma. Red hair/fair skin, a phenotype due to loss of function polymorphism in the MC1R gene (melanocortin one receptor gene), is significantly implied in melanoma. The loss of function variants of this gene is also linked to an increased risk of PD [48]. Mutation of Parkin, a tumor suppressor gene located on chromosome 6q25-27, causes young-onset PD [49]. Similarly, the loss of heterozygosity in the chromosome6q25-q27 region is also noted in human cancers, including melanoma [50]. These studies imply the possible genetic association with the simultaneous occurrence of melanoma and PD.

An important role of physicians caring for PD or melanoma is to inform their patients adequately on relevant knowledge of the disease and their associations. They should be advised on skin protection from ultraviolet (UV) rays, especially in lighter skin tones. Physicians should also make the patients cautious about any new or changing pigmented skin lesions [5]. PD and melanoma associations specifically require an acknowledgement among dermatologists, neurologists, oncologists, ophthalmologists, and general practitioners who are directly involved in these patients' care.

## Conclusions

The condition of the skin in patients of PD is often trivialized and overlooked. The primary purpose of our review article was to elucidate the dermatological disorders in PD and explore the linkage in their pathophysiology, genetics, and immunology, focusing on any role of the nervous system in the skin. This knowledge gives a better ground for dermatologists, neurologists, oncologists, and general physicians to advise and care better for their patients and improve their quality of life. Future surgical/nonsurgical treatment options for PD may positively or negatively affect the skin disorders associated with PD. Therefore, further research is needed to establish the robust cause of the increased prevalence of skin disorders in PD.
